# Establishment and Application of a Quantitative Lipemia Index as a Novel Indicator for Assessing Lipemic Interference in Clinical Chemistry

**DOI:** 10.1002/jcla.70378

**Published:** 2026-10-06

**Authors:** Haifeng Xie, Shuhua Bao, Pengcheng Tong, Peng Li, Zhe Shen, Jinlong Wei

**Affiliations:** ^1^ Laboratory Medicine Center Hangzhou Traditional Chinese Medicine (TCM) Hospital Affiliated to Zhejiang Chinese Medical University Hangzhou China; ^2^ Diagnostics Division Beckman Coulter (China) Co., Ltd. (North China Region) Shenyang China; ^3^ Department of Laboratory Medicine Tonghua Central Hospital Tonghua China; ^4^ Department of Laboratory Medicine Central Hospital of Dalian University of Technology Dalian China

**Keywords:** lipemia index, lipemic interference, method validation, triglyceride (TG)

## Abstract

**Background:**

Lipemia is a common pre‐analytical interference in clinical chemistry. Triglyceride (TG) concentration is commonly used as a surrogate indicator for lipemic interference, but its predictive reliability is limited by heterogeneity in lipoprotein particle composition. This study aimed to establish and validate a quantitative Lipemia index (LI) assay for direct measurement of serum turbidity, compare its predictive value with TG, and evaluate its clinical applicability through reference interval estimation and correlation with interference deviations in native patient samples.

**Methods:**

The LI assay was developed on a Beckman Coulter AU5800 analyzer by measuring Δ*A* (*A*_660_–*A*_800 nm_). Performance was validated per CLSI guidelines: precision (EP05‐A3), detection capability (EP17‐A2), linearity (EP06‐ED2), method comparison (Passing‐Bablok regression, Bland–Altman analysis), reference interval (EP28‐A3c), and dilution recovery (EP07‐A2). LI's predictive value for lipemic interference was compared to TG using deviations in total protein assays (single‐ vs. double‐reagent methods).

**Results:**

The LI assay demonstrated precision with CV ≤ 2.23% and linearity (0.05–30.87 mmol/L TG‐equivalent). It correlated strongly with the reference TG method (*ρ* = 0.998, *p* < 0.0001). In predicting assay interference, LI showed a closer correlation with total protein deviations than did TG (*r*_s_ = 0.822 vs. 0.770). A reference interval of 0.093–0.240 was established for healthy adults. Dilution recovery (90%–110%) confirmed accuracy.

**Conclusions:**

The quantitative LI assay provides a standardized, clinically validated measure of lipemic interference aligning more directly with the optical nature of clinical chemistry testing than TG measurement. These findings support its analytical validity, utility as a predictor of turbidity interference, and clinical applicability.

## Graphical abstract

A rigorously validated quantitative lipemia index (LI) assay was established for direct serum turbidity measurement. This standardized LI metric demonstrated significantly stronger correlation with lipemic interference (*r*_s_ = 0.822) than conventional triglyceride concentration (*r*_s_ = 0.770), consistent with its direct measurement of optical turbidity. The assay provides a reliable, CLSI‐validated tool for informing specimen acceptability decisions in routine clinical laboratories.

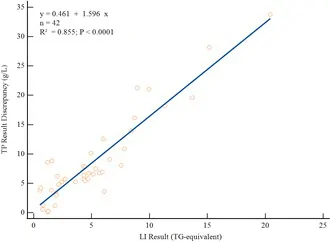

## 1 Introduction

The presence of high‐concentration lipoprotein particles induces light scattering, leading to turbidity in serum or plasma. This common phenomenon causes significant interference with test results on clinical chemistry analyzers through various mechanisms [1]. For instance, light scattering due to lipemia profoundly impacts turbidimetric and nephelometric assays across the entire visible spectrum (300–700 nm) [2]. Although non‐fasting blood sampling is the primary cause of lipemia, factors such as chronic alcohol consumption, diabetes, renal disease, total parenteral nutrition, and certain medications can also contribute [3].

To evaluate assay susceptibility to lipemic interference, in vitro diagnostic manufacturers predominantly rely on adding fat emulsions (e.g., Intralipid) to samples. Traditionally, the triglyceride (TG) concentration in these supplemented samples serves as a surrogate for turbidity‐induced interference. However, relying solely on TG concentration to predict turbidity‐induced interference in patient samples is unreliable [4]. This limitation stems from fundamental differences in the physicochemical properties of TG‐containing particles within synthetic emulsions compared to those found in naturally occurring lipemic clinical samples [4, 5]. Consequently, directly measuring serum or plasma turbidity is recognized as a more robust and clinically relevant indicator. Therefore, establishing precise and quantitative methods for turbidity assessment is crucial.

Although most clinical chemistry platforms offer automated assessment of serum indices (including hemolysis, icterus, and lipemia), these indices are typically reported in categorical or semi‐quantitative formats (e.g., +, ++, +++, ++++) rather than as continuous numerical values. Although they have significant potential utility [2], current limitations hinder their effectiveness for this purpose. Insufficient transparency in manufacturers' specifications for the turbidity‐measuring lipemia index (LI), the inability of many analyzers to provide fully quantitative serum index results, and the lack of publicly available correlation data between lipemia indices and triglyceride concentrations are critical gaps [6]. As a result, most laboratories rely on categorical or semi‐quantitative lipemia indices that are platform‐specific, poorly harmonized, and not directly comparable across different analyzers. In addition, the widely adopted practice of using triglyceride concentration as a surrogate for lipemic interference is undermined by the inherent heterogeneity of lipoprotein particles, which differ in size, composition, and light‐scattering efficiency—a limitation that complicates the establishment of universal thresholds for sample rejection or result flagging. The hypothesis underlying the present study is that a directly measured, fully quantitative turbidity index, standardized and traceable to a reference material, would correlate more closely with actual interference effects than triglyceride concentration. Unlike manufacturer‐specific serum indices, the proposed LI assay is designed to be transferable across analytical platforms through explicit specification of all analytical parameters (sample volume, wavelength configuration, calibration mode, and measurement point), thereby providing a harmonized approach to lipemic interference assessment. We therefore aimed to establish a rigorously validated quantitative LI assay that accurately reflects serum or plasma turbidity, to compare its predictive performance with TG as a surrogate for lipemic interference using a controlled analytical model (single‐ vs. double‐reagent total protein assays), and to evaluate its clinical applicability by establishing a reference interval in healthy adults and assessing its correlation with interference deviations in native patient samples.

## 2 Materials and Methods

### 2.1 Materials

All experiments were performed using serum samples. The LI reagent used was sodium chloride solution (0.9%; Kelun Pharma Co. Ltd., Sichuan, China). For analytical validation experiments (precision, linearity, detection capability, and dilution recovery), artificial lipemic samples were prepared by spiking 0.9% sodium chloride solution (Kelun Pharma Co. Ltd., Sichuan, China) with Intralipid emulsion (20%; Fresenius Kabi SSPC, Wuxi, China). Intralipid is a commercially available intravenous lipid emulsion composed of 20% soybean oil, 1.2% egg yolk phospholipids, and 2.25% glycerin in water for injection, with a mean particle size of approximately 330 nm. Here, Intralipid served as a stable, homogeneous, and traceable turbidity reference material, following its established use in CLSI guidelines and by in vitro diagnostic manufacturers for lipemic interference testing. 0.9% sodium chloride solution was used both as the LI reagent and as the diluent for artificial lipemic samples because it is chemically inert, has negligible absorbance at the measurement wavelengths (660/800 nm), and closely approximates the osmotic properties of serum, thereby providing a stable and reproducible matrix for turbidity assessment without confounding optical interference. Although Intralipid does not fully replicate the particle heterogeneity of naturally occurring lipemic sera, its optical properties provide a reproducible basis for assessing the analytical performance of the turbidity‐based LI assay.

The following commercial assay kits were employed: a single‐reagent total protein test kit (Biuret method; 540/700 nm; Single‐point End Method; Reebio Biotechnology Co. Ltd., Ningbo, China); a double‐reagent total protein test kit (Biuret method; 540/700 nm; Two‐point End Method; Beckman Coulter Inc., California, USA); a triglyceride test kit (Glycerol‐3‐phosphate oxidase/Peroxidase (GPO‐POD) method; Saike Biotechnology Co. Ltd., Ningbo, China).

Clinical chemistry analysis was performed using a Beckman Coulter AU5800 analyzer (Beckman Coulter Inc., California, USA).

### 2.2 Analytical Method

The LI assay was performed on the Beckman Coulter AU5800 system. For each test, 10 μL of sample was combined with 70 μL of reagent (R1). The reaction mixture was incubated at 37°C for 2 min. Absorbance was then measured at primary and secondary wavelengths of 660 and 800 nm, respectively (Δ*A* = *A*_660_–*A*_800 nm_). Measurements utilized the endpoint method with linear calibration mode and a positive reaction direction (increasing absorbance). Based on these established reaction parameters, the LI assay was configured as a new, independent test on the analyzer. The complete analyzer application parameters for the LI assay on the AU5800 system are provided in Table S1.

### 2.3 Preparation of Calibrators and Quality Controls

The LI calibrator (target: 10 mmol/L TG‐equivalent) was selected to cover clinically relevant hyperlipidemic concentrations [7, 8] and align with the linear mid‐range of the analytical measurement interval (AMI) per Clinical & Laboratory Standard Institute (CLSI) EP06‐ED2 [9]. This concentration reflects pathological levels requiring intervention (TG > 10 mmol/L) as defined in clinical guidelines and the lipid interference literature [3, 7, 8], while avoiding rare excessive values (< 0.5% prevalence for TG > 30 mmol/L) [3, 7]. The calibrator was generated through aseptic dilution of Intralipid emulsion (20%) with 0.9% sodium chloride solution, establishing the relationship between the absorbance difference (Δ*A*_660–800 nm_) and the LI scale (TG concentration of the calibrator). Its final TG concentration was verified via the enzymatic method on the Beckman Coulter AU5800 analyzer.

Four quality control (QC) materials, representing distinct turbidity levels, were similarly prepared with target TG concentrations of 1 mmol/L (Level 1), 8 mmol/L (Level 2), 12 mmol/L (Level 3), 16 mmol/L (Level 4). The actual turbidity of each QC level was confirmed through absorbance measurements.

The stability of the Intralipid‐based calibrator and QC materials under the defined storage conditions was verified through pre‐experimental testing. These tests indicated that freeze–thaw cycles alter the physical properties of lipid particles in both Intralipid‐based and patient‐derived materials; therefore, freeze–thaw was avoided. Calibrators were prepared freshly for each calibration event. All lipemic materials (calibrator and QCs) were stored at 4°C until analysis. Storage and handling protocols followed Vrtaric et al. [10] (addressing emulsion stability) and ISO Guide 80:2014 [11] (guidelines for QC material preparation). QC materials were analyzed in each run and remained consistently within acceptance limits throughout the study period, providing practical assurance of calibration reliability. Prior to analysis, all samples were homogenized by repeated inversion for 10 times.

A single‐point calibration with zero‐intercept was used for the LI assay. This approach was adopted because the assay measures a physical property—turbidity‐induced light scattering—rather than a biochemical reaction, and the reagent blank (0.9% NaCl) has negligible absorbance. The validity of this calibration strategy was subsequently confirmed by the linearity verification across the analytical measurement range (Section 2.4.3).

### 2.4 Analytical Performance Validation

#### 2.4.1 Detection Capability (LoB, LoD, LoQ)

The limit of blank (LoB), limit of detection (LoD), and limit of quantification (LoQ) for the LI assay were evaluated according to the classical approach described in the CLSI EP17‐A2 [12].

LoB and LoD determination: For LoB, five independent replicate preparations of a blank matrix (0.9% sodium chloride solution) were used. For LoD, five independent replicate preparations of a low‐concentration (turbidity) LI sample were used (prepared at approximately 5 times the preliminary LoB estimate). Each of the five blank preparations and each of the five low‐concentration preparations was measured in duplicate on each of seven separate days, yielding 70 measurements per sample type (*n* = 70 for LoB and *n* = 70 for LoD). LoB and LoD were derived via nonparametric percentile method (EP17‐A2, Chapter 5.3.3).

LoQ determination: The LoQ was established through repeated analysis of four aliquots from three low‐concentration lipemic samples (TG‐equivalent concentrations: 0.05, 0.075, and 1 mmol/L). Testing was performed over five independent days with three replicate measurements per aliquot daily (total *n* = 60). The LoQ was validated to meet the following acceptance criteria per FDA Bioanalytical Method Validation Guidance (2018) [13]: (a) Relative standard deviation (RSD) of repeated measurements ≤ 20%; (b) Mean measured value within ±20% of the nominal concentration.

#### 2.4.2 Precision and Method Comparison

Precision evaluation: Precision (CV%) was evaluated per CLSI EP05‐A3 [14]. Four in‐house QCs (TG: 1; 8; 12; 16 mmol/L; see Calibrator Preparation section) representing turbidity gradients were analyzed over 20 non‐consecutive days (2 runs/day, duplicates/run; *n* = 80/level).

Method comparison: Method comparison study was performed using a panel of at least 50 lipemic samples prepared by spiking Intralipid emulsion into a base matrix (0.9% sodium chloride solution) to generate a wide turbidity range; ultimately, 56 samples were included in the analysis. TG concentrations in these samples were quantified using the enzymatic triglyceride assay. The LI values were compared to TG using Passing‐Bablok regression (nonparametric, robust to outliers) to estimate slope and intercept. Bland–Altman analysis was applied to assess bias across the measurement range.

#### 2.4.3 Linearity

The Linearity of the LI assay was validated according to the Clinical and Laboratory Standards Institute (CLSI) guideline EP06‐ED2, Chapter 3 [9]. A high‐turbidity sample was prepared by diluting 20% Intralipid emulsion with 0.9% sodium chloride solution (diluent matrix) to target a TG concentration of approximately 30 mmol/L, exceeding the assay's anticipated upper limit of quantification and challenging the upper boundary of AMI. This high‐turbidity sample was then subjected to proportional serial dilution with the same diluent matrix to generate a linearity panel comprising 11 concentration levels (theoretical relative concentrations: 0% [diluent matrix only], 10%, 20%, …, 100% [undiluted high‐turbidity sample]).

TG concentration was used as the assigned reference value because it served as the traceable standard for calibrator assignment and is measured by an internationally standardized enzymatic method. Each concentration level was measured for LI and TG concentrations (as assigned true values) in duplicate on the AU5800 analyzer. Mean measured LI values and TG concentrations were calculated for each level. Linearity was evaluated using least‐squares linear regression analysis (using no intercept in the model), with the TG concentrations as the independent variable (*x*‐axis) and the mean measured LI as the dependent variable (*y*‐axis). The acceptance criterion required: (a) a slope between 0.95 and 1.05; (b) visual inspection of the linearity and residual plot indicating no significant systematic deviation; (c) the absolute value of the percentage residual [(Observed − True)/True × 100%] at each concentration level less than 7.5% (1/2 total allowable error, TEa [15]).

#### 2.4.4 Dilution Recovery

To assess the accuracy of the LI assay in highly lipemic samples that require dilution before measurement, dilution recovery was evaluated according to CLSI EP07‐A2 [16] using two lipemic patient samples with elevated TG concentrations, including one sample with a TG concentration of approximately 30 mmol/L to simulate severe hyperlipemia. Each sample was independently diluted 1:2 and 1:4 with 0.9% NaCl solution, with three separate aliquots prepared per dilution factor (totaling 6 aliquots per sample). The undiluted sample was measured in triplicate to establish baseline LI result (acceptance criterion: CV ≤ 5%), while each diluted aliquot was measured once. Recovery rates were calculated using the formula: [(mean diluted LI × dilution factor)/mean undiluted LI] × 100%. The study required all recovery rates to fall within 90%–110%, with ≤ 15% deviation between replicate aliquots at each dilution level, ensuring both accuracy and precision of the dilution protocol for lipemic samples.

### 2.5 Reference Interval Determination

The reference interval for the LI assay was developed in accordance with CLSI EP28‐A3c guidelines [17] using 140 de‐identified serum samples collected from clinically healthy adults (age ≥ 16 years) who underwent routine biochemical testing at Hangzhou TCM Hospital Affiliated to Zhejiang Chinese Medical University between February and April 2026. Fasting blood samples were collected in the morning, allowed to clot naturally at room temperature, and then centrifuged at 1200 × g for 10 min to obtain serum for clinical chemistry analysis. Reference individuals were confirmed to have no history of chronic metabolic diseases (e.g., diabetes, renal dysfunction), lipid‐modifying medication use, or lifestyle factors affecting lipid metabolism (alcohol intake > 20 g/day or smoking > 10 cigarettes/day), nor abnormal immunoglobulin levels. All samples demonstrated normal lipid profiles (low‐density lipoprotein cholesterol, high‐density lipoprotein cholesterol, TG, and total cholesterol within clinically validated ranges) and were visually inspected to exclude hemolysis, icterus, and lipemia. To minimize preanalytical variability, samples were analyzed in a single batch on the Beckman Coulter AU5800 system without prior freeze–thaw cycles, following the analytical protocol described in the Analytical Method section. Outlier exclusion was pre‐specified in the study protocol using Tukey's method with a threshold of 1.5 × IQR (interquartile range), consistent with CLSI EP28‐A3c guidelines for nonparametric reference interval estimation [17]. After excluding identified outliers, reference limits (2.5th and 97.5th percentiles) were determined nonparametrically and statistically validated using MedCalc (v23.0.2).

### 2.6 Comparative Assessment of Lipemic Interference Prediction

Given the unavailability of a reference method and the impracticality of ultracentrifugation for lipid removal, we employed a comparative strategy utilizing paired single‐ and double‐reagent total protein assays (both biuret‐based, 540/700 nm absorbance) to quantify lipid turbidity interference. Compared to the single‐reagent method, the double‐reagent method employs a two‐point end measurement, which corrects for background absorbance interference by subtracting the sample blank reading from the final absorbance. Total protein was selected as the model analyte because the biuret method is a spectrophotometric endpoint assay that is particularly susceptible to turbidity interference, and the availability of both single‐ and double‐reagent formats allowed us to isolate turbidity‐induced interference from other analytical variations. This provides a clean experimental model for comparing the predictive performance of LI and TG without confounding factors. The protocol comprised two phases:

Phase 1: Inter‐method calibration: following CLSI EP9‐A2 guidelines [18], we performed method comparison using 40 paired measurements from non‐lipemic samples (visually clear sera; serum indices negative for lipemia; triglyceride concentrations within the reference interval), covering a broad range of total protein concentrations to establish a Deming regression equation between the single‐ and double‐reagent assays to ensure the consistency of test results between the two reagents.

Phase 2: Interference Quantification: a cohort of 42 native lipemic serum samples (TG range: 4.20–42.92 mmol/L; mean: 15.89 mmol/L; median: 11.22 mmol/L) was analyzed through: (a) TG quantification (enzymatic GPO‐POD assay); (b) LI measurement; (c) Duplicate total protein measurements via both single‐ and double‐reagent assays. These samples were randomly selected from routine clinical chemistry specimens submitted to Hangzhou TCM Hospital. Samples were included if triglyceride concentration exceeded 4.0 mmol/L, as concentrations below this level generally do not produce clinically significant lipemic interference [4, 8], with selection aimed to cover a broad range of lipemic levels encountered in routine practice. Samples with abnormal immunoglobulin levels were not excluded, as the objective of Phase 2 was to evaluate LI performance under real‐world conditions where such co‐existing conditions may occur. Samples were not pooled into predefined lipemia strata, as the study objective was to assess the correlation between LI and interference deviations across the full spectrum of lipemia encountered in routine practice. All samples were characterized by automated serum indices (hemolysis, icterus, lipemia) on the AU5800 analyzer, supplemented by visual inspection and, where available, bilirubin results, as severe lipemia may interfere with automated index readings. Samples with visible clots, insufficient volume, or significant hemolysis/icterus were excluded. All samples were processed within 4 h of collection, stored at 4°C prior to analysis, and homogenized by repeated inversion (10 times) before measurement. No freeze–thaw cycles were applied. The complete dataset of these 42 samples, including TG concentration, LI value, and corrected TP deviation, is provided in Table S2.

Single‐reagent results were adjusted using the Deming regression equation to correct for systematic bias. The corrected deviation—defined as the absolute difference between the Deming‐corrected single‐reagent TP result and the corresponding raw double‐reagent TP result—served as the primary metric for quantifying lipid interference, with larger values indicating greater analytical bias attributable to turbidity. Given the non‐normal distribution of the variables (Shapiro–Wilk test, all *p* < 0.001), Spearman's rank‐order correlation coefficients (*r*_s_) were computed for the following pairs: (a) deviations vs. TG concentrations; (b) deviations vs. LI values; (c) TG concentrations vs. LI values. Finally, Meng's *Z*‐test (*α* = 0.05) was utilized to statistically compare the relative strength of the correlation observed in (a) with that in (b) (Data S1).

### 2.7 Data Analysis

All statistical analyses were performed in accordance with CLSI guidelines and standard statistical methodologies. Precision evaluation via analysis of variance (ANOVA) was conducted using Microsoft Excel (Microsoft Corp., USA), adhering to CLSI EP05‐A3 guidelines [14]. For reference interval determination and method comparison analyses (including Bland–Altman plot analysis, Deming regression, Passing & Bablok regression, and least‐squares linear regression), MedCalc (version 23.0.2; MedCalc Software Ltd., Ostend, Belgium) was employed. Additionally, Meng's *Z*‐test for comparing dependent correlation coefficients was performed using R statistical software (version 4.5.1) with the cocor package (version 1.1.4) [19].

## 3 Results

### 3.1 Calibration Curve Establishment

Following the standardized procedures described in Section 2.2 (Analytical method) and 2.3 (Calibrator preparation) sections, we prepared a lipemic calibrator with a TG concentration of 9.8 mmol/L. The calibration curve demonstrated a linear relationship between turbidity absorbance (*x*‐axis, measured as Δ*A*_660–800 nm_) and derived lipemia index (*y*‐axis, expressed as TG‐equivalent concentration in mmol/L), defined by the equation: *y* = 30.25*x* (where *x =* Δ*A*_660–800 nm_, *y =* lipemia index in TG‐equivalent, mmol/L).

### 3.2 Precision and Accuracy

As described in Section 2.4.2 (Precision and method comparison), the LI method demonstrated precision across clinically relevant TG concentrations with total CV of 1.00%, 1.36%, 1.64%, and 2.23% at target concentrations of 1.00, 8.00, 12.00, and 16.00 mmol/L TG‐equivalent (Table 1).

**TABLE 1:** Precision performance of the quantitative lipemia index method.

Precision estimates (target TG concentration)	Level 1 (1.00 mmol/L)	Level 2 (8.00 mmol/L)	Level 3 (12.00 mmol/L)	Level 4 (16.00 mmol/L)
Actual mean concentration (TG‐equivalent mmol/L)	0.99	7.94	11.85	15.99
Repeatability (CV%)	1.47	0.81	0.99	1.30
Between‐day precision (CV%)	0.33	0.26	0.89	0.39
Within‐laboratory precision (CV%)	1.64	1.00	2.23	1.36

*Note:* Within‐laboratory precision, total CV (*n* = 80 per level).

Abbreviation: CV, coefficient of variation.

Method comparison analysis of 56 samples using Passing‐Bablok regression revealed strong agreement between the LI assay and the reference GPO‐POD assay, evidenced by a slope of 1.004 (95% CI: 0.990–1.021), an intercept of −0.0399 mmol/L (95% CI: −0.072 to −0.018), and a very high Spearman's correlation coefficient (*ρ* = 0.998, *p* < 0.0001; 95% CI: 0.996–0.999) (Figure 1A).

**FIGURE 1:**
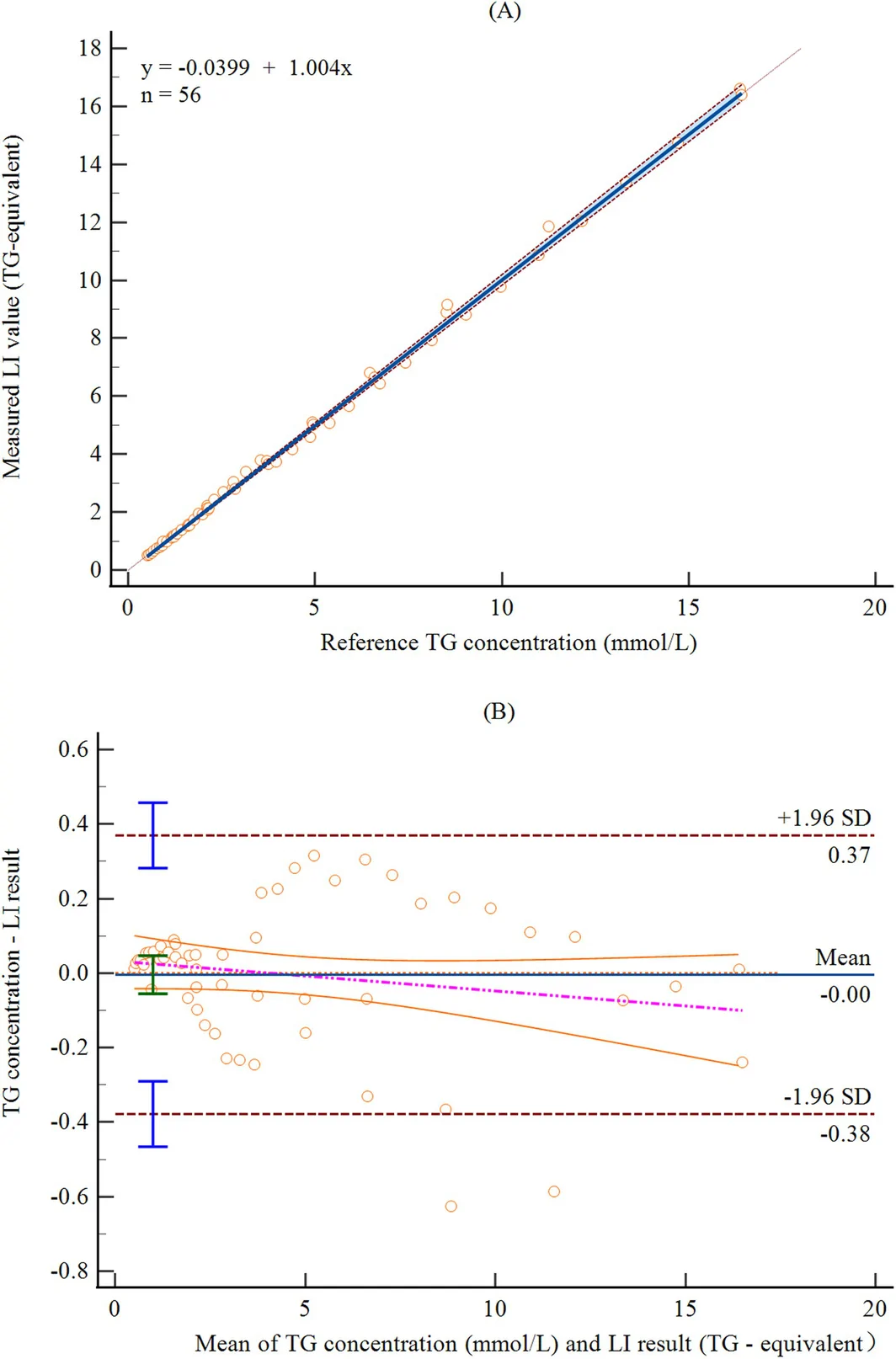
Method comparison between the quantitative lipemia index assay and the reference triglyceride assay (*n* = 56). (A) Passing–Bablok regression analysis showing the correlation between lipemia index (LI) values (TG‐equivalent, mmol/L) and triglyceride (TG) concentrations (mmol/L). The solid line represents the regression line, and the dashed line represents the line of identity. The regression equation is as follows: Slope = 1.004 (95% CI: 0.990–1.021), intercept = −0.0399 mmol/L (95% CI: −0.072 to −0.018). Spearman's correlation coefficient: *ρ* = 0.998 (95% CI: 0.996–0.999, *p* < 0.0001). (B) Bland–Altman plot illustrating the agreement between the LI assay and the TG method. The solid line indicates the mean difference (−0.0042 mmol/L), and the dashed lines represent the 95% limits of agreement (−0.378 to 0.370 mmol/L). No statistically significant proportional bias was observed (*p* = 0.871).

Bland–Altman analysis further confirmed minimal systematic bias, with a mean difference of −0.0042 mmol/L between methods (95% limits of agreement: −0.378 to 0.370 mmol/L) and no statistically significant deviation (*p* = 0.871) (Figure 1B).

### 3.3 Detection Capability and Linearity

As described in Sections 2.4.1 and 2.4.3, the LI method exhibited robust analytical performance across the evaluated range. Nonparametric statistical methods were employed for limit determination due to non‐normally distributed measurement variability (Shapiro–Wilk test, *p* < 0.05) [20].

The LoB was 0.009 mmol/L TG equivalents, with a LoD of 0.039 mmol/L TG equivalents. The low‐concentration LI sample used for LoD determination was prepared at a final concentration of 0.039 mmol/L TG‐equivalent (approximately 4.3× the LoB, consistent with the intended 5× target). The validated LoQ was 0.05 mmol/L TG equivalents, meeting acceptance criteria for precision (relative standard deviation, RSD = 19.32%) and accuracy (mean bias = −12% of nominal concentration). Given that this concentration represents a very low turbidity level—well below clinically relevant lipemic thresholds—the analytical performance at the LoQ is considered practically satisfactory.

Method linearity was verified over the clinically relevant range of 0–30.87 mmol/L TG equivalents. A strong correlation was observed between LI‐derived TG equivalents and reference TG concentrations as the regression equation: *y* = 0.984*x* (*n* = 11, slope 95% CI: 0.964–1.004, *p* < 0.0001) (Figure 2 and Table 2). As shown in Table 2, the percentage deviations at all concentration levels were within the predefined allowable deviation from linearity (ADL) of ±7.5%. These results validate an AMI of 0.05–30.87 mmol/L TG equivalents for serum turbidity quantification.

**FIGURE 2:**
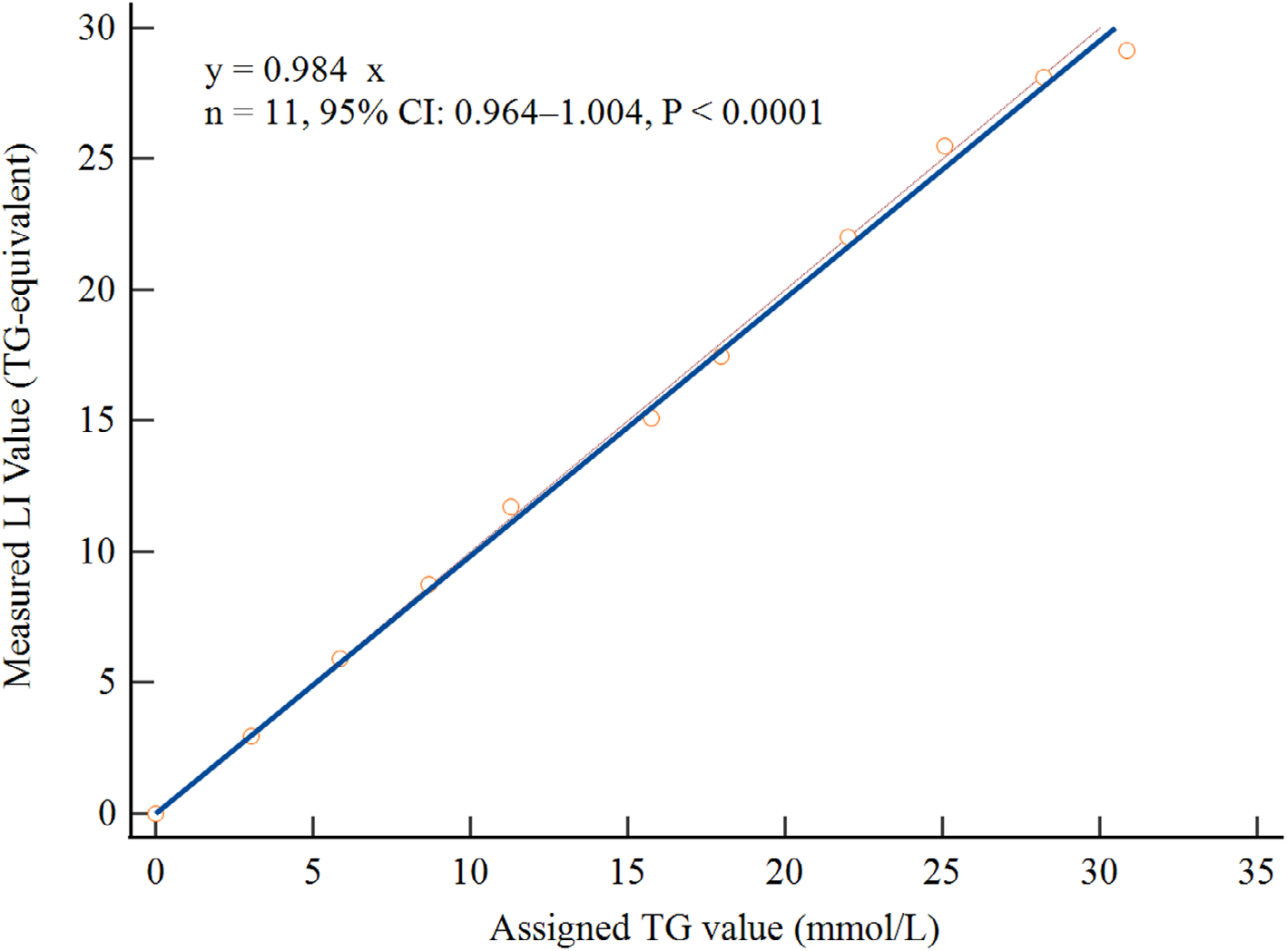
Linearity of the quantitative lipemia index assay. The plot shows the correlation between measured lipemia index (LI) values (TG‐equivalent, mmol/L) and expected triglyceride (TG) concentrations (mmol/L) across the analytical measurement range of 0.05–30.87 mmol/L. Each point represents the mean of duplicate measurements at 11 concentration levels prepared by serial dilution of a high‐turbidity sample. The solid line represents the linear regression line (forced through the origin), and the dashed lines represent the 95% confidence intervals. The regression equation is: *Y* = 0.984*x* (*n* = 11, 95% CI for slope: 0.964–1.004, *p* < 0.0001).

**TABLE 2:** Linearity of the quantitative lipemia index (LI) assay.

Sample	RC	LI value (TG‐equivalent, mmol/L)	Assigned value (mmol/L)	Deviation (mmol/L)	Deviation (%)	Within ADL = ±7.5%
1	1.00	29.16	30.87	−1.71	−5.5	YES
2	0.91	28.13	28.23	−0.10	−0.4	YES
3	0.81	25.48	25.06	0.42	1.7	YES
4	0.71	22.02	22.01	0.01	0.1	YES
5	0.61	17.45	17.96	−0.51	−2.8	YES
6	0.51	15.09	15.75	−0.66	−4.2	YES
7	0.37	11.71	11.30	0.41	3.6	YES
8	0.28	8.75	8.69	0.06	0.7	YES
9	0.19	5.92	5.86	0.06	1.0	YES
10	0.10	2.97	3.04	−0.07	−2.3	YES
11	0	0.0011	0.00	−0.0011	−0.1	YES

Abbreviations: ADL, allowable deviation from linearity; RC, relative concentration; RC, assigned value/30.87.

### 3.4 Reference Interval

As described in Section 2.5 (Reference interval determination), after excluding 8 individuals who meet the pre‐defined exclusion criteria (see Flowchart, Figure 1, for details), 132 initial samples remained. From these, 6 samples (4.55%) were flagged as outliers by Tukey's method and excluded, leaving a final cohort of 126 samples for analysis. This rate was consistent with the expected 5% threshold for the 1.5 × IQR cutoff. The reference interval for the LI assay was established using samples from 126 healthy donors (57 males, 69 females; mean age 35.55 ± 8.26 years). Subsequently, the nonparametric percentile method (CLSI EP28‐A3 [17]) was applied via MedCalc software, yielding a double‐sided reference limit from 0.093 (90% CI: 0.063–0.102) to 0.240 (90% CI: 0.210–0.258). Sex‐stratified reference interval data are provided in Table S3.

Age‐stratified analysis suggested a decreasing trend in the upper reference limit in older age groups (≥ 50 years), though these findings should be interpreted with caution given the limited sample size in these subgroups and the non‐significant age terms in the fitted model (*p* > 0.05 for all age coefficients). The complete age‐stratified data are provided in Table S4. Further investigation in larger, age‐diverse cohorts is warranted to confirm potential age‐associated variations.

### 3.5 Dilution Recovery

As described in Section 2.4.4 (Dilution recovery), dilution recovery testing was conducted on two highly lipemic samples (Sample 1: 19.35 mmol/L TG; Sample 2: 27.60 mmol/L TG) to validate the assay's performance. Both undiluted samples demonstrated precision with CV of 0.96% and 0.17%, respectively, well below the 5% acceptance criterion. Across all tested dilution levels, recovery rates consistently fell within the 90%–110% target range, with inter‐replicate variations ≤ 15% (Table 3). These results confirm the accuracy and precision of the dilution protocol.

**TABLE 3:** Dilution recovery of the quantitative lipemia index assay.

Sample	Baseline conc (TG‐equivalent, mmol/L)	Undiluted CV (%)	Dilution factor	Recovery (%)^a^	Aliquot deviation (%)	Max deviation (%)	Within ≤ 15% criteria
1	19.35	0.96	2×	104.40 ± 1.19	+0.22, −1.31, +1.09	1.31	YES
			4×	103.76 ± 2.79	+0.10, −2.88, +2.78	2.88	YES
2	27.60	0.17	2×	100.14 ± 0.59	+0.29, −0.68, +0.40	0.68	YES
			4×	100.24 ± 0.84	+0.69, −0.93, +0.24	0.93	YES

*Note:* SD calculated from three independent measurements at each dilution level.

Abbreviations: Conc, concentration; CV, coefficient of variation.

^a^ Recovery data expressed as mean ± SD (*n* = 3 aliquots per dilution factor).

### 3.6 Comparative Analysis of Indicators for Lipemic Interference

As described in Section 2.6 (Comparative assessment of lipemic interference prediction), the Deming regression validated a robust relationship between single‐reagent and double‐reagent total protein assays: *y* = 2.076 [95% CI: 0.866–3.286] + 1.019*x* [95% CI: 1.000–1.038] (*r* = 0.998, 95% CI: 0.997–0.999; *x*: single‐reagent TP [g/L]; *y*: double‐reagent TP [g/L], Figure 3). Spearman's rank‐order correlation was used to assess the relationships between each indicator and the absolute deviations in TP measurements. LI showed a correlation of *r*_s_ = 0.822 (95% CI: 0.691–0.901) with absolute TP deviations, compared with *r*_s_ = 0.770 (95% CI: 0.609–0.870) for TG (Figure 4A,B). Linear regression analyses revealed that LI explained 85.5% of the variance in TP deviations (deviation = 0.461 + 1.596 × LI; *R* ^2^ = 0.855; *p* < 0.0001), while TG explained 72.0% (deviation = −1.604 + 0.682 × TG; *R* ^2^ = 0.720; *p* < 0.0001). The difference between the two correlation coefficients was not statistically significant (Δ*r*_s_ = 0.052; Meng's *Z*‐test, *p* = 0.422; Zou's 95% CI: −0.079 to 0.201). Bootstrap resampling confirmed this finding (95% CI: −0.140 to 0.249). Sensitivity analysis excluding the single most influential observation (identified by Cook's distance) yielded similar results (Δ*r*_s_ = 0.095; *p* = 0.092; see Table S5 for full results). Although LI and TG concentrations were correlated (*r*_s_ = 0.699, 95% CI: 0.501–0.827), the observed dispersion suggests that TG alone may not reliably predict lipemic interference in all cases (Figure 4C).

**FIGURE 3:**
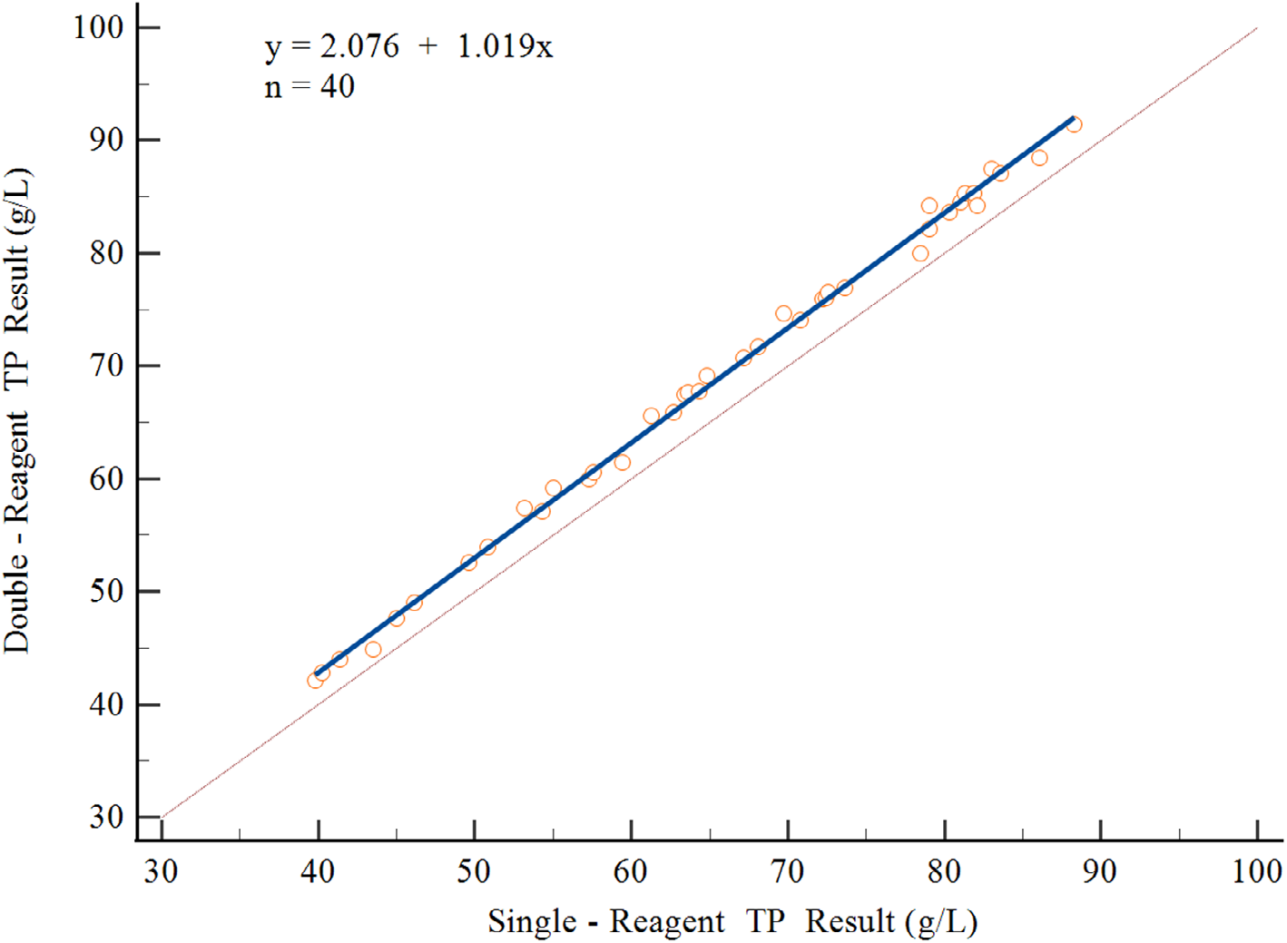
Deming regression analysis comparing single‐reagent and double‐reagent total protein assays (*n* = 40). The solid line represents the Deming regression line, and the dashed line represents the line of identity. The regression equation is: *Y* = 2.076 + 1.019*x* (95% CI for intercept: 0.866–3.286; 95% CI for slope: 1.000–1.038). Deming regression correlation coefficient: *R* = 0.998 (95% CI: 0.997–0.999). *x*: Single‐reagent total protein (TP) (g/L); *y*: Double‐reagent TP (g/L).

**FIGURE 4:**
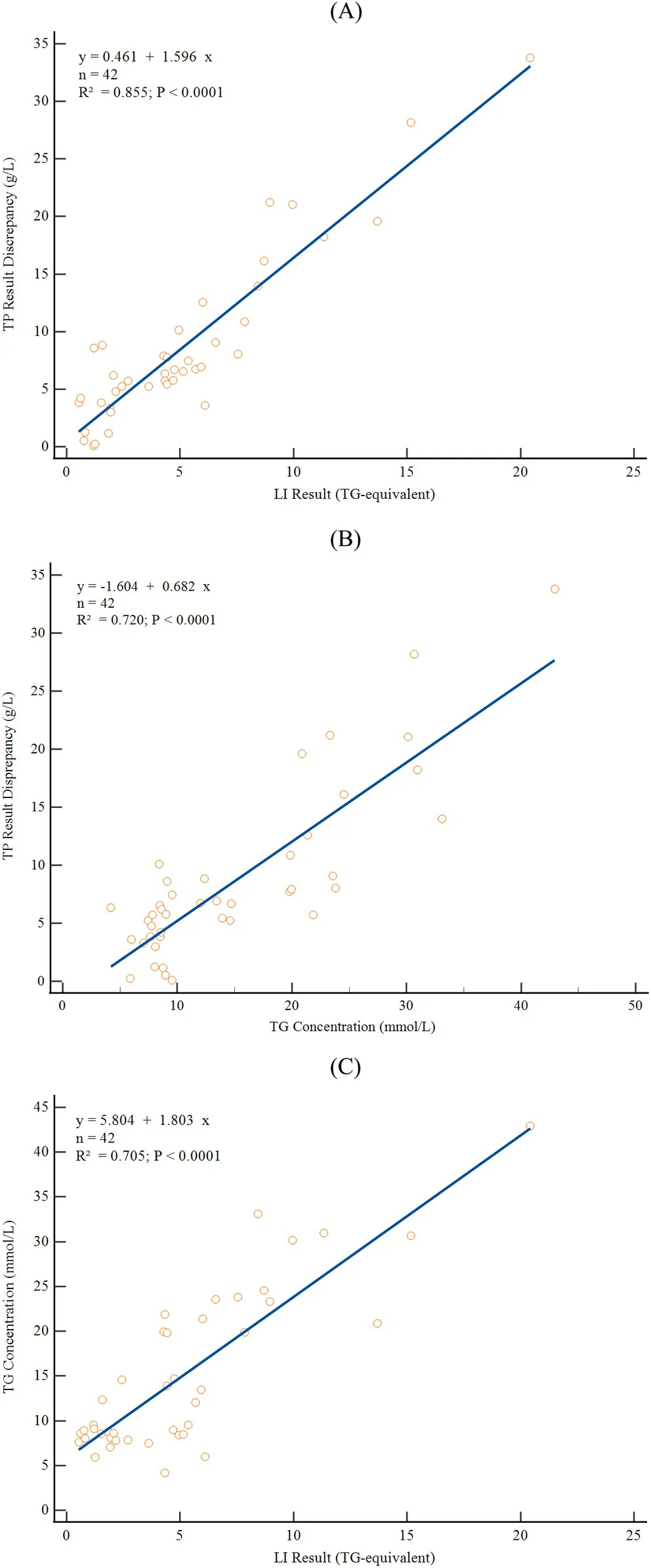
Comparative predictive performance of the lipemia index and triglyceride for assessing lipemic interference (*n* = 42). (A) Correlation between lipemia index (LI) values (mmol/L TG‐equivalent) and absolute deviations in total protein (TP) measurements (g/L). (B) Correlation between TG concentrations (mmol/L) and absolute deviations in TP measurements (g/L). (C) Correlation between LI values and TG concentrations (mmol/L). Solid lines represent linear regression fits: (A) deviation = 0.461 + 1.596 × LI (*R* ^2^ = 0.855, *p* < 0.0001); (B) deviation = −1.604 + 0.682 × TG (*R* ^2^ = 0.720, *p* < 0.0001); (C) TG = 5.804 + 1.803 × LI (*R* ^2^ = 0.705, *p* < 0.0001). Spearman's rank‐order correlation coefficients (*r*_s_) with 95% confidence intervals are shown in each panel. (A) *r*_s_ = 0.822 (95% CI: 0.691–0.901); (B) *r*_s_ = 0.770 (95% CI: 0.609–0.870); (C) *r*_s_ = 0.699 (95% CI: 0.501–0.827). All *p* values < 0.0001. The difference between the correlations in (A) and (B) was not statistically significant (Δ*r*_s_ = 0.052; Meng's *Z*‐test, *p* = 0.422; Zou's 95% CI: −0.079 to 0.201).

## 4 Discussion

This study establishes and validates a novel quantitative LI assay based on Δ660/800 nm spectrophotometry. The assay exhibits precision with CV < 2.23% across a wide linear range (0.05–30.87 mmol/L TG‐equivalent), addressing the critical need for standardized turbidity quantification. Most notably, it demonstrated a closer correlation with total protein assay deviations in lipemic samples compared to the conventional use of TG concentration as a surrogate marker for lipemic interference—a practice commonly adopted in manufacturer interference claims and reagent package inserts (*r* = 0.822 vs. 0.770; Δ*r*_s_ = 0.052), suggesting its practical utility as a more direct predictor of lipemic interference in optical‐platform settings, although the difference between the two correlations was not statistically significant (*p* = 0.422; see Results 3.6).

Our findings resolve longstanding limitations in lipemia interference management. Although TG concentration has been conventionally adopted as a surrogate for turbidity—particularly in manufacturer interference claims; its poor correlation with actual turbidity is well documented [4]. It is worth noting that many contemporary analyzers already measure turbidity spectrophotometrically and report a lipemia index. However, these indices are typically semi‐quantitative (categorical scales, e.g., +, ++, +++, ++++) and platform‐specific, lacking full analytical validation and traceability. The LI assay proposed here addresses these limitations by providing a fully quantitative, CLSI‐validated, and transferable measure that can be directly aligned with manufacturer interference thresholds expressed in TG‐equivalent units, thereby bridging the gap between semi‐quantitative instrument indices and reagent‐based interference claims. This approach is grounded in the fundamental principle that turbidity is influenced by lipoprotein particle heterogeneity—a concept highlighted by both Pozharski [21] and Nikolac [8]. Their work demonstrates that variations in lipoprotein size and composition (e.g., chylomicrons vs. very low‐density lipoprotein, VLDL) differentially affect light scattering, explaining the poor correlation between TG concentration and actual turbidity. The closer correlation between LI and total protein assay deviations (*r*_s_ = 0.822) compared to TG (*r*_s_ = 0.770) highlights LI's practical relevance for detecting lipemia‐induced interference in optical‐platform‐based testing. Furthermore, by employing TG‐equivalent units, the quantitative LI method can be directly applied to traditional lipemic interference specifications listed in reagent package inserts without modification. Notably, manufacturer‐provided interference thresholds—including instrument‐specific L‐index claims—are not always consistent with empirically determined cutoffs, as demonstrated by Knezevic et al. [22] for L‐index claims on the Roche cobas platform; however, reagent manufacturers' interference specifications expressed in TG concentrations remain the most widely used reference for laboratories. The use of TG‐equivalent units does not imply that TG is an optimal surrogate for turbidity. Rather, this convention is adopted for practical compatibility: reagent package inserts and interference claims are almost universally expressed in TG concentrations, and expressing LI in the same units allows laboratories to directly compare LI‐based assessments with existing manufacturer‐provided thresholds without the need for unit conversion or recalibration.

Beyond its immediate utility for interference assessment, the quantitative LI assay opens doors to broader clinical applications. It not only serves as a more directly relevant indicator for assessing lipemic interference compared to triglyceride levels but may also aid in identifying patients with potential lipid metabolism disorders. The establishment of a reference interval (0.093–0.240) for healthy individuals provides a clinically actionable threshold to identify pathological lipemia and supports further lipid testing in suspected cases. Sex‐stratified analysis (Table S3) showed that the 97.5th percentile for males (0.244) was slightly higher than that for females (0.207), while the 2.5th percentile for males (0.103) was slightly higher than that for females (0.088). Despite these numerical differences, the combined reference interval (0.093–0.240) remained broadly applicable to both sexes in this predominantly young adult cohort. However, the subgroup sample sizes (male: *n* = 57; female: *n* = 69) do not meet the CLSI EP28‐A3c minimum recommendation for formal reference interval establishment (*n* ≥ 120 per subgroup), and the age distribution was skewed toward younger adults. Age‐stratified analysis (Table S4) suggested a decreasing trend in the upper reference limit in older age groups (≥ 50 years). However, the limited sample size in these subgroups and the non‐significant age coefficients in the fitted model (*p* > 0.05 for all age terms) preclude definitive conclusions. Therefore, larger studies with more balanced sex and age distributions are needed to determine whether sex‐ or age‐specific reference intervals are required.

Furthermore, quantitative measurement of the lipemia index may extend its clinical applications beyond lipemia assessment, as emerging studies have proposed it as a potential indicator for monoclonal gammopathies [21, 23]. It is worth noting that free glycerol interference—which can artificially elevate triglyceride measurements in standard one‐step enzymatic assays (pseudohypertriglyceridemia)—is typically negligible in routine clinical practice and does not materially affect the interpretation of our findings [24, 25]. Thus, quantitative LI measurements, interpreted alongside TG values, could serve as valuable markers for identifying glycerol metabolism disorders. Given the established correlation between turbidity of suspensions and particle size [5], the combined use of quantitative lipemia index and triglyceride concentration may enable initial phenotyping of hyperlipidemia. As indicated by Ercan et al. [26], quantitative LI measurements could enable the establishment of more precise reflex testing protocols, such as targeted ultracentrifugation or method‐specific interference mitigation strategies.

Before interpreting the clinical implications of our findings, it is important to clarify the distinct roles of Intralipid in this study. For analytical validation experiments (precision, linearity, detection capability, and dilution recovery), Intralipid was employed as a stable, homogeneous, and traceable turbidity reference material, consistent with its established use in CLSI guidelines and by in vitro diagnostic manufacturers for lipemic interference testing [7, 10]. The purpose of this approach was to assess the analytical performance of the LI assay—specifically, whether the method could reliably quantify turbidity across a defined measurement range—rather than to biologically replicate the full heterogeneity of endogenous lipemic particles. We acknowledge that Intralipid does not perfectly mirror the particle size distribution or light‐scattering behavior of naturally occurring lipemic sera at equivalent triglyceride concentrations [27]; however, this distinction does not undermine the validity of the analytical validation. Rather, it underscores the need for complementary evaluation in authentic clinical specimens. In this regard, our analyses performed on 42 genuine lipemic patient samples (Figure 4) confirmed that LI maintains a strong correlation with interference deviations in real‐world settings (*r*_s_ = 0.822, 95% CI: 0.691–0.901), supporting the translational relevance of the assay beyond the Intralipid‐based validation framework.

Although the selected wavelengths (660/800 nm) are theoretically positioned to minimize absorbance from hemoglobin and bilirubin, we acknowledge that lipemic samples in clinical practice frequently co‐occur with hemolysis or icterus, particularly in emergency settings. Published spectral data demonstrate that hemoglobin displays strong absorbance at 415, 540, and 570 nm, with minimal absorbance above 600 nm [28]. Consistently, Beckman Coulter AU‐series analyzers utilize 660 nm as one of the wavelengths for hemolysis index assessment (Table 2) Simundic et al. [28], supporting the theoretical robustness of the LI assay against hemoglobin interference. Similarly, bilirubin absorbance decreases substantially above 500 nm and is minimal at 660 nm. Although these theoretical considerations suggest that the LI assay is relatively robust to co‐existing hemolysis and icterus, direct experimental verification—particularly at clinically relevant hemoglobin indices (e.g., 1+ to 3+) and bilirubin concentrations (e.g., ≥ 200 μmol/L)—remains a priority for future validation. Such studies would further strengthen the practical applicability of the LI assay in routine laboratory settings, especially in emergency and critical care populations where co‐occurring interferences are common.

However, several limitations must be acknowledged. A methodological consideration worth emphasizing is the shared optical basis between the LI assay and the interference surrogate used in this study. The single‐ minus double‐reagent total protein deviation, employed here as a practical proxy for lipemic interference in the absence of ultracentrifugation, is itself derived from optical absorbance measurements on the same analytical platform as the LI assay. Consequently, the correlation between LI and this deviation metric (*r*_s_ = 0.822) is partly attributable to their common optical measurement framework rather than reflecting a purely independent predictive relationship. We acknowledge this as an inherent limitation of our study design. Nevertheless, we argue that this does not diminish the translational value of the LI assay: in routine clinical chemistry, interference assessment is invariably performed on optical platforms, and reagent manufacturers' interference claims are likewise validated using optical methods. Therefore, a turbidity index that directly quantifies the optical phenomenon responsible for interference is inherently more aligned with the practical reality of laboratory testing than a chemical concentration (TG) that only indirectly reflects it. The LI assay should be viewed as a complement to, rather than a replacement for, TG measurement, with its primary value lying in providing direct, real‐time turbidity information that TG cannot offer. The reference interval was derived from a single‐center cohort, with a relatively young adult population (mean age 35.6 ± 8.3 years), and the LI assay was validated on a single analytical platform (with the manufacturer affiliation disclosed in the Conflict of Interest statement); therefore, validation in more age‐diverse and geographically diverse cohorts, multi‐center and multi‐platform validation will be important next steps to confirm broader applicability. In the dilution recovery experiment, each diluted aliquot was measured once rather than in replicate; this approach was intentionally adopted to reflect routine clinical practice, where samples requiring dilution are typically measured once after dilution. The satisfactory recovery rates (all within 90%–110%) and low inter‐aliquot variability (≤ 2.88%, Table 3) provide confidence in the robustness of the results despite the single‐measurement design. The TG concentrations of the native lipemic samples ranged from 4.20 to 42.92 mmol/L; therefore, the performance of the LI assay in samples with TG levels below 4.20 mmol/L—including the clinically relevant 4.0 mmol/L threshold—was not evaluated. The inclusion criterion was set at ≥ 4.0 mmol/L, and 4.20 mmol/L was the closest available sample meeting this threshold. Future studies should include a broader range of TG concentrations, particularly at lower lipemic levels. In addition, the proposed LI assay was not directly compared with the semi‐quantitative lipemia index provided by the AU5800 analyzer, as the two indices operate on fundamentally different measurement scales (categorical vs. continuous). Such a comparison would not be methodologically meaningful without a common reference framework. Future studies may explore the relationship between the LI assay and manufacturer‐provided lipemia indices once standardized quantitative measurements become available on clinical chemistry platforms. Although the complete analytical specifications are explicitly defined—including sample‐to‐reagent ratio (10 μL/70 μL), incubation parameters (37°C, 2 min), and dual‐wavelength configuration (660/800 nm)—enabling transferability to other photometric analyzers, prospective multi‐platform validation will further support harmonized implementation [6, 29].

Beyond its immediate utility for interference assessment, this study provides a methodological framework for standardizing lipemia index measurement across different analytical platforms. Current manufacturer‐dependent implementations of serum indices suffer from methodological heterogeneity—including varying wavelengths, calibration approaches, and result reporting formats—which limits their clinical utility and precludes establishment of universal thresholds [29, 30]. Notably, these indices are typically reported in semi‐quantitative categorical scales (e.g., −, +, ++), whereas the proposed LI assay provides a continuous, fully quantitative measure expressed in TG‐equivalent units. This distinction allows direct alignment with reagent manufacturers' interference claims—which are conventionally stated in TG concentrations—and enables cross‐platform comparability that categorical indices cannot offer. By defining a complete set of analytical parameters validated against CLSI guidelines, and by establishing a traceable calibrator with TG‐equivalent units compatible with existing reagent package inserts, this work offers a blueprint for harmonized LI implementation. The explicit specifications provided herein can serve as a reference for both clinical laboratories seeking to implement quantitative LI testing and for manufacturers aiming to align their serum indices methodologies. In summary, the adoption of this standardized quantitative LI assay provides a reliable surrogate for direct turbidity measurement, offering a more clinically relevant and practice‐oriented metric for assessing lipemic interference and informing clinical decisions regarding specimen acceptability.

In light of these limitations, collective standardization efforts by instrument manufacturers and clinical laboratories—informed by this study's analytical framework—are imperative to establish robust LI metrics. This harmonization will transcend platform‐specific limitations, empowering clinically actionable decisions and catalyzing mechanistic discoveries in lipid metabolism. Further investigation is warranted to explore its extended utility in lipoprotein phenotyping, detection of glycerol metabolism disorders, and importantly, the validation of age‐ and sex‐specific reference intervals in larger cohorts, as well as the establishment of standardized LI thresholds that could be integrated into automated reflex testing algorithms across different analytical platforms to fully realize its potential beyond interference assessment. The potential role of LI in detecting non‐spectrophotometric interference mechanisms—such as volume displacement, analyte partitioning, and sample heterogeneity—also warrants further investigation. In addition, future studies should validate the LI assay across a broader panel of clinically relevant analytes to further confirm its generalizability for lipemic interference assessment.

## Author Contributions

H.X., S.B., P.T., and J.W. designed the experiments. H.X., S.B., and P.T. performed the experiments and collected the data. H.X., S.B., and J.W. analyzed and validated the data. P.L. provided technical support for the Beckman Coulter AU5800 platform. H.X., S.B., P.T., P.L., Z.S., and J.W. drafted and revised the manuscript. All authors have accepted responsibility for the entire content of this manuscript and approved submission.

## Funding

This work was supported by the Key Discipline Fund of Clinical Laboratory Medicine, Hangzhou TCM Hospital Affiliated to Zhejiang Chinese Medical University [Grant Number 2026YJZD13]. The funder had no role in the conceptualization, design, data collection, analysis, decision to publish, or preparation of the manuscript.

## Ethics Statement

This study was conducted in accordance with the Declaration of Helsinki (as revised in 2013). Ethical approval was granted by the Ethics Committee of Hangzhou TCM Hospital Affiliated to Zhejiang Chinese Medical University on January 26, 2026 (approval No. 2026KLL036).

## Consent

Informed consent was obtained from all individuals included in this study.

## Conflicts of Interest

Peng Li is an employee of Beckman Coulter (China) Co. Ltd., the manufacturer of the AU5800 clinical chemistry analyzer used in this study. His contribution to this work was made in a personal capacity as a co‐author and was not part of his assigned work duties. The company had no role in the study design, data collection, analysis, interpretation, manuscript preparation, or the decision to submit. All other authors declare no competing interests.

## Supporting information


**Data S1:** jcla70378‐sup‐0001‐DataS1.docx.

**Table S1:** Beckman Coulter AU5800 analyzer application parameters for the quantitative lipemia index (LI) assay.

**Table S2:** Raw data of the 42 native lipemic samples used for correlation analysis between LI, TG concentration, and corrected TP deviation.

**Table S3:** Sex‐stratified reference interval for the quantitative lipemia index (LI) assay.

**Table S4:** Age‐stratified reference limits for the quantitative lipemia index (LI) assay (2.5th and 97.5th percentiles by age group).

**Table S5:** Spearman rank‐order correlations, Meng's *Z*‐test, and sensitivity analysis for comparing LI and TG in predicting lipemic interference.

## Data Availability

The data that support the findings of this study are available on request from the corresponding author. The data are not publicly available due to privacy or ethical restrictions.
