# Supplementary material for: Establishment and Application of a Quantitative Lipemia Index as a Novel Indicator for Assessing Lipemic Interference in Clinical Chemistry
Source: J Clin Lab Anal. 2026 Oct 6:e70378. Online ahead of print. doi: 10.1002/jcla.70378 (PMC13639763; doi:10.1002/jcla.70378)
Supplement: Supplementary file 1 [file JCLA-9999-e70378-s002.docx]

**Supplementary Table 4.** Age-stratified reference limits for the quantitative lipemia index (LI) assay (2.5th and 97.5th percentiles by age group).

| **Age group (years)** | **n** | **2.5th percentile** | **97.5th percentile** |
| --- | --- | --- | --- |
| 20–24 | 7 | 0.1015 | 0.2556 |
| 25–29 | 16 | 0.0968 | 0.2102 |
| 30–34 | 20 | 0.1067 | 0.2443 |
| 35–39 | 27 | 0.0869 | 0.2154 |
| 40–44 | 26 | 0.1009 | 0.1968 |
| 45–49 | 13 | 0.1166 | 0.2061 |
| 50–54 | 6 | 0.1131 | 0.1788 |
| 55–59 | 2 | — | — |
| 60–64 | 0 | — | — |
| 65–69 | 1 | — | — |

**Footnote:** Age groups with n < 5 (55–59 years: n=2; 60–64 years: n=0; 65–69 years: n=1) are not shown due to insufficient sample size for meaningful percentile estimation. The mean age of the cohort was 35.6 ± 8.3 years, with the majority of participants distributed between 25 and 49 years.
