# Supplementary material for: Establishment and Application of a Quantitative Lipemia Index as a Novel Indicator for Assessing Lipemic Interference in Clinical Chemistry
Source: J Clin Lab Anal. 2026 Oct 6:e70378. Online ahead of print. doi: 10.1002/jcla.70378 (PMC13639763; doi:10.1002/jcla.70378)
Supplement: Supplementary file 2 [file JCLA-9999-e70378-s003.docx]

**Supplementary Table 1.** Beckman Coulter AU5800 analyzer application parameters for the quantitative lipemia index (LI) assay.

| **Parameter** | **Setting** |
| --- | --- |
| Test Name | Lipemia Index (LI) |
| Sample Volume | 10 µL |
| R1 Volume | 70 µL |
| R2 Volume | — |
| Total Reaction Volume | 80 µL |
| Wavelength (Primary) | 660 nm |
| Wavelength (Secondary) | 800 nm |
| Reaction Direction | Positive |
| Assay Type | Endpoint |
| Calibration Mode | Linear through zero |
| Calibration Points | 1 (single-point calibrator) |
| Measurement Point | 2 minutes after incubation |
| Incubation Temperature | 37°C |
| Incubation Time | 2 minutes |
| Sample Cup Type | Standard cuvette |
| Reagent Position | As specified in analyzer configuration |

**Footnote:** The LI assay was configured as a new, independent test on the Beckman Coulter AU5800 analyzer based on the parameters described in the Methods section (subsection 2.2). Additional instrument-specific settings not listed here follow the manufacturer's default configuration for endpoint absorbance assays.
