# Supplementary material for: Establishment and Application of a Quantitative Lipemia Index as a Novel Indicator for Assessing Lipemic Interference in Clinical Chemistry
Source: J Clin Lab Anal. 2026 Oct 6:e70378. Online ahead of print. doi: 10.1002/jcla.70378 (PMC13639763; doi:10.1002/jcla.70378)
Supplement: Supplementary file 3 [file JCLA-9999-e70378-s004.docx]

**Supplementary Table 3.** Sex-stratified reference interval for the quantitative lipemia index (LI) assay.

| **Sex** | **n** | **Mean** | **SD** | **Median** | **Min** | **Max** | **2.5th percentile (90% CI)** | **97.5th percentile (90% CI)** |
| --- | --- | --- | --- | --- | --- | --- | --- | --- |
| Male | 57 | 0.1597 | 0.0387 | 0.1573 | 0.1003 | 0.2556 | 0.1032 (0.1003–0.1154) | 0.2443 (0.2032–0.2556) |
| Female | 69 | 0.1430 | 0.0316 | 0.1387 | 0.0625 | 0.2183 | 0.0879 (0.0625–0.1015) | 0.2066 (0.1893–0.2183) |
| Combined | 126 | 0.1498 | 0.0361 | 0.1427 | 0.0625 | 0.2579 | 0.0930 (0.0630–0.1020) | 0.2400 (0.2100–0.2580) |

**Footnote:** Subgroup sizes (male: n=57; female: n=69) do not meet the CLSI EP28-A3c minimum recommendation for formal reference interval establishment (n≥120 per subgroup). Values are presented for descriptive purposes only. Combined reference interval values are from the main analysis (nonparametric method).
