# Supplementary material for: Establishment and Application of a Quantitative Lipemia Index as a Novel Indicator for Assessing Lipemic Interference in Clinical Chemistry
Source: J Clin Lab Anal. 2026 Oct 6:e70378. Online ahead of print. doi: 10.1002/jcla.70378 (PMC13639763; doi:10.1002/jcla.70378)
Supplement: Supplementary file 4 [file JCLA-9999-e70378-s005.docx]

**Supplementary Table 5**. Spearman rank-order correlations, Meng's Z-test, and sensitivity analysis for comparing LI and TG in predicting lipemic interference.

| **Analysis** | **n** | **r_s_**  **(LI vs. AbsDev)** | **r_s_**  **(TG vs. AbsDev)** | **r_s_**  **(LI vs. TG)** | **Δr_s_** | **95% CI** | **P value** |
| --- | --- | --- | --- | --- | --- | --- | --- |
| **Spearman correlation (complete data)** | 42 | 0.822 (0.691–0.901) | 0.770 (0.609–0.870) | 0.699 (0.501–0.827) | 0.052 | −0.079 to 0.201^a^ | 0.422^a^ |
| **Bootstrap validation** | 42 | — | — | — | — | −0.140 to 0.249^b^ | — |
| **Sensitivity analysis (excluding the single most influential observation)**^c^ | 41 | 0.862 | 0.767 | 0.752 | 0.095 | −0.015 to 0.244^a^ | 0.092^a^ |

^a^ Based on Meng's Z-test with Zou's confidence interval for dependent correlations (cocor R package).

^b^ Based on 5000 bootstrap resamples (percentile method).

^c^ The single most influential observation (sample #38) was identified as having the largest Cook's distance.

**Abbreviations:** LI, lipemia index; TG, triglyceride; AbsDev, absolute deviation; CI, confidence interval; n, sample size; r_s_, Spearman's rank-order correlation coefficient; Δr_s_, difference between the two Spearman correlation coefficients.
