# Supplementary material for: Establishment and Application of a Quantitative Lipemia Index as a Novel Indicator for Assessing Lipemic Interference in Clinical Chemistry
Source: J Clin Lab Anal. 2026 Oct 6:e70378. Online ahead of print. doi: 10.1002/jcla.70378 (PMC13639763; doi:10.1002/jcla.70378)
Supplement: Supplementary file 5 [file JCLA-9999-e70378-s006.docx]

\# Supplementary Code

\# Spearman correlation, bootstrap validation, and influence diagnostics

\# for the comparison of LI vs. TG in predicting lipemic interference

\#

\# This code reproduces the analytical workflow for transparency and reproducibility.

\# Final statistical results reported in the manuscript were calculated using

\# MedCalc (v23.0.2) for correlation coefficients and R for Meng's Z-test,

\# bootstrap, and influence diagnostics.

library(cocor)

library(boot)

\# Data: X = LI, Y = TG, Z_raw = raw deviation (single - double reagent TP)

X <- c(8.6976, 1.9334, 4.7529, 4.3261, 13.6848, 2.0770, 0.7728, 4.4343,

11.3232, 4.2653, 5.6766, 4.3217, 8.4301, 1.9171, 6.5711, 4.7035,

1.8432, 4.4296, 3.6132, 5.1495, 0.8042, 5.3646, 7.8533, 7.5529,

5.9196, 2.4378, 1.5272, 1.1978, 1.5653, 0.6193, 20.4306, 2.1668,

4.9387, 2.6989, 0.5317, 8.9479, 1.1812, 6.1033, 1.2533, 5.9888,

15.1696, 9.9457)

Y <- c(24.54, 8.09, 14.68, 21.87, 20.91, 8.63, 8.94, 19.83, 30.98, 19.95,

12.03, 4.20, 33.12, 7.04, 23.56, 9.0, 8.78, 13.90, 7.45, 8.48,

8.02, 9.52, 19.88, 23.79, 13.46, 14.59, 8.52, 9.09, 12.35, 8.59,

42.92, 7.76, 8.43, 7.83, 7.62, 23.34, 9.53, 5.98, 5.9, 21.36,

30.68, 30.15)

Z_raw <- c(16.1278, 2.9863, 6.6994, 5.7421, 19.607, 6.2131, 0.5097, 7.7462,

18.2392, 7.9196, 6.7401, 6.3376, 13.9806, 3.3294, 9.082, 5.7546,

1.1417, 5.4297, 5.2474, 6.5557, 1.2466, 7.4626, 10.8719, 8.0417,

6.9435, 5.2485, 3.8534, 8.5942, 8.8282, 4.2096, 33.7862, 4.7787,

10.1108, 5.7408, 3.8307, 21.2181, -0.1064, 3.6073, -0.2216, 12.5737,

28.1783, 21.0421)

\# Absolute deviation

Z_abs <- abs(Z_raw)

df <- data.frame(X, Y, Z_abs)

\# 1. Shapiro-Wilk normality test (as referenced in the manuscript)

shapiro.test(X)

shapiro.test(Y)

shapiro.test(Z_abs)

\# 2. Spearman rank correlations (as reported in the manuscript)

r_xz <- cor(df$X, df$Z_abs, method = "spearman")

r_yz <- cor(df$Y, df$Z_abs, method = "spearman")

r_xy <- cor(df$X, df$Y, method = "spearman")

r_xz

r_yz

r_xy

r_xz - r_yz

\# 3. Meng's Z-test for dependent correlations

cocor.dep.groups.overlap(

r.jk = r_xz,

r.jh = r_yz,

r.kh = r_xy,

n = nrow(df)

)

\# 4. Bootstrap 95% CI for Δr (5000 resamples)

boot_delta <- function(data, indices) {

d <- data[indices, ]

r1 <- cor(d$X, d$Z_abs, method = "spearman")

r2 <- cor(d$Y, d$Z_abs, method = "spearman")

r1 - r2

}

set.seed(123)

boot_out <- boot(df, boot_delta, R = 5000)

boot.ci(boot_out, type = "perc")

\# 5. Influence diagnostics (leave-one-out)

delta_loo <- numeric(nrow(df))

for (i in 1:nrow(df)) {

d <- df[-i, ]

r1 <- cor(d$X, d$Z_abs, method = "spearman")

r2 <- cor(d$Y, d$Z_abs, method = "spearman")

delta_loo[i] <- r1 - r2

}

cooks_d <- (delta_loo - mean(delta_loo))^2 / var(delta_loo)

\# Identify the single most influential observation

which.max(cooks_d)

\# 6. Sensitivity analysis excluding the single most influential observation

df_excl <- df[-which.max(cooks_d), ]

r_xz_excl <- cor(df_excl$X, df_excl$Z_abs, method = "spearman")

r_yz_excl <- cor(df_excl$Y, df_excl$Z_abs, method = "spearman")

r_xy_excl <- cor(df_excl$X, df_excl$Y, method = "spearman")

r_xz_excl

r_yz_excl

r_xz_excl - r_yz_excl

cocor.dep.groups.overlap(

r.jk = r_xz_excl,

r.jh = r_yz_excl,

r.kh = r_xy_excl,

n = nrow(df_excl)

)
